# A structural equation model of the relationship among occupational stress, coping styles, and mental health of pediatric nurses in China: a cross-sectional study

**DOI:** 10.1186/s12888-022-04061-4

**Published:** 2022-06-21

**Authors:** Yating Zhou, Xiaoli Guo, Huaying Yin

**Affiliations:** Children’s Hospital of Chongqing Medical University, Chongqing Key Laboratory of Childhood Nutrition and Health, National Clinical Research Center for Child Health and Disorders, Ministry of Education Key Laboratory of Child Development and Disorders, Chongqing, 400014 China

**Keywords:** Pediatric nurses, Occupational stress, Coping styles, Mental health

## Abstract

**Background:**

Pediatric nurses experience a wide rang of stressful events at work every day, which can trigger a lot of emotional responses. The objectives of this study were mainly to explore the potential interrelationships of occupational stress, coping styles and mental health among pediatric nurse.

**Methods:**

A total of 381 pediatric nurses from Chongqing, China were recruited in this cross-sectional study. We performed this study based on a questionnaire survey that contained the Chinese Perceived Stress Scale (CPSS), Simplified Coping Style Questionnaire and Symptom-Checklist 90(SCL-90).

**Results:**

The pediatric nurses reported having health risk stress(HRS) was 54.3%, and nurses with different medical professional titles, style of coping and profiles of mental health had significantly different occupational stress levels *(P* < *0.01)*. And with the application of the Spearman correlation analysis and Structural Equation Modelling were revealed a significant relationship among occupational stress, coping style and mental health. The positive coping style had a negative direct predictive effect on occupational stress (*β* = *-0.499, P* < *0.01*) and mental health symptoms (β = -0.115, *P* < 0.01), negative coping styles had positive predictive effect on occupational stress (β = 0.185, *P* < 0.01) and mental health symptoms (β = 0.205, *P* < 0.01). Occupational stress had significant impact on mental health symptoms (*β* = 0.416, *P* < 0.01), and it was played a part of mediating effect between coping style and mental health.

**Conclusion:**

These findings demonstrated significant associations between occupational stress, coping style and mental health in pediatric nurses, and this SEM model highlighted that the potential prediction effects of occupational stress and coping styles for mental health and the mediated effect of occupational stress between coping style and mental health, which we believe facilitates the understanding of these associations. This model should be useful in the formulation of strategies to improve mental health level for this population.

## Background

Occupational stress, also known as stress in the work place, is described as a pattern of physiological, emotional cognitive and behavioral responses that occur when workers are presented with work demands not matched to their knowledge, skills, or abilities and which challenge their ability to cope [1]. Occupational stress is recognized worldwide as a major problem facing healthcare workers [2]. Epidemiological studies [3–5] conducted in different countries have shown that healthcare workers were at a higher risk of suffering from occupational stress, which directly or indirectly affected the quality and safety of medical/clinical service. Therefore, the health-related issues being encountered by the healthcare workers themselves are being increasingly recognized.

Nursing has been acknowledged to be a stressful occupation with a high prevalence of distress and worked-related stress [6], notably in pediatric settings as the structure and service is very particularity and complexity. Pediatric nurses experience repetitive stress when dealing with sick children and their emotional and desperate parents, which may be extra stressful compared to other general nurses [7]. Moreover, some nurses’ job in the pediatric includes inter alia working under specific circumstances and frequent exposure to longer work shifts, human suffering, grief, death and events which are far beyond usual human experience. Various indicators such as higher incidence of work discontent, turnover rate, insomnia, musculoskeletal diseases, more frequent post-traumatic stress disorders (PTSD), and even suicide suggest [8–10] that pediatric nurses are exposed to higher risk as compared to the general working population and other healthcare workers. For those working in the business environment, burnout and poor job productivity are signs of occupational stress [11]. In addition, studies have shown that some confounding variables do exist when studying stress-related influencing factors. For example, age, which means that experience over time could allow older nurses to have lower stress and hormonal or immunologic responses to adverse events [12]. Recognition of stress in nurses by this way could provide a new idea for us to promoting effective stress management strategies in the future and help combat stress-prone caregivers.

The topic of occupational stress has been given considerable attention as an important reference guide of psychological health. Chronic excessive continuous stress may worsen their mental health conditions, causing difficulty in the ability to function at work. Nursing staff, mainly in public hospitals, face poor working conditions, which can be associated with mental health problems, such as depression, and anxiety. An survey about the 1-year prevalence of mood disorders in Dutch showed that citizens with a high income was 3.0%, and the 1-year prevalence of anxiety disorders was 6.0% vs 29% and 24% respectively for physicians [9]. However, the prevalence of anxiety and mood disorders in Chinese adults according to the National Mental Health Survey could be as high as 7.4% and 7.6% [13], respectively. And for nurses, the prevalence of both depression and anxiety disorders were 43.4% and 61.7% [14, 15]. Consequently, the increasing numbers of nurses feel frustrated and burned out in their job, and related behavioral health issues have drawn public attention. An important task for research is to determine the variables that might protect nurses’ mental health against occupational stress.

Lazarus and Folkman [16] defined coping as constantly changing cognitive and behavioral efforts to manage specific internal/external demands that are appraised as exceeding the resources of the person. Coping mechanisms are categorized as problem solving and emotionally focused coping. Lin et al. [17] found that the effective utilization of coping mechanisms interferes with the level of stress and depression experienced. Moreover, a study of hospital nurses in Australia and New Zealand found that the avoidance type of coping strategy was significantly associated with physical and mental health [18]. This means that a stress level can be mediated if an individual administers the effective coping strategies to handle the stressors. However, there is a paucity of studies examining these variables in Middle East countries, and the majority of the previous studies were conducted with nurses as follows: emergency, ICU, psychiatric, community [17, 19–21]. Little is known about pediatric nurses in mainland China, and an association among occupational stress, coping strategy and mental health is not unclear.

Under the current opening-two child-policy in China, the working conditions of pediatric nurses have become exhausting and highly stressful in recent years due to heavy workloads and litigation, extended working hours and high rates of workplace violence, a lack of control over work, and tense nurse-patient-parent relationships [22–25]. Moreover, many graduates of Medical University were unwilling to work in children’s hospital [26]. Hu WL et al. [27] found that there were only 1.3 pediatric staffs (0.6 doctors and 0.7 nurses) in per ten thousand Chinese people. It means that critical situations need to be faced is the shortage of pediatric nurses. As our previously studies described [28] that the prevalence of health related stress (HRS) in pediatric staffs was 70%, which having higher stress risk than the general population. Thus, further understanding occupational stress and coping variables that may relate most closely to mental health among pediatric nurses is necessary. The quantitative studies already conducted in this area used classical statistical procedures assessing straightforward relationships, such as linear or logistic regression. However, the association between all of these factors is probably more complex. Structural equation modeling (SEM) is a statistical procedure that allows testing non- straightforward relationships and is therefore well suited to the management of cross-sectional data for inferential purposes. These models enable the simultaneous fit of several multiple linear regressions and the variables present in the regressions may be either observable or latent [29].

The present study aimed to explore the relationships of occupational stress, coping styles and mental health among pediatric nurses in our institute, and the potential modifying effect of occupational stress on this association.

## Methods

### Participants

The cross-sectional study was conducted in Chongqing, China from December 2019 to February 2020, and a convenience method was used to recruit participants from a tertiary specialized children’s hospital. All pediatric nurses from different departments, including Internal Medicine, Surgery and Outpatients, Emergency, and Intensive Care Unit (ICU). In each department, we invited all nurses who satisfied the inclusion criteria to participate this study. The inclusion criteria were: 1)qualified nurses with registration; 2)more than 6 months of working experience in at least one pediatric department; 3)no history of mental illnesses; 4)consent to participate in the study. Nurses were excluded if they were intern nurses or receiving standardized training, or if they worked less than 6 months in pediatric department.

### Participant recruitment

The research was conducted according to the guidelines stated in the Declaration of Helsinki, and ethical approval was obtained from an Institutional Review Board. Prior to data collection, we explained to every participant the purposes and significance of the survey before obtaining their written informed contents. And we also informed their right to withdraw from the study at any time or choose not to answer questions about their experience. Participants participated in the study voluntarily and anonymously and their information were kept strictly confidential and only used for this research.

### Measures

#### Demographic variables

Demographic variables included age, gender, educational level, marital status, title, department, and length of employment.

#### Occupational stress

A Chinese version of Perceived Stress Scale (CPSS) was used to assess the nurses’ occupational stress. It had been reported as having good reliability and validity in Chinese populations [30]. The questionnaire contains 14 items of two scales. Responses to all the items were scored as 0 meaning never and 4 meaning always. The higher scores, the higher level of stress. Respondents with a total score above 25 were considered as Health Related Stress (HRS). The Cronbach alpha coefficients of the scale and the two subscales named “the tension” and “the sense of loss” were 0.75,0.79, and 0.85, respectively.

#### Coping style

The style of coping strategies was assess by the Simplified Coping Style Questionnaire(SCSQ) [31]. It was a validated instrument of 20 statements within positive copying (12 items) and negative copying (8 items). Responses to all the items were scored from 0 to “Never do” and 3 to “always do”. The higher the scores, the more likely this copying style is to be used by the participants. The scale’s alpha was 0.90, and the positive and negative copying subscales’ alpha were 0.89 and 0.78, respectively.

#### Mental health

A 90-item Symptom Checklist 90 (SCL-90) [32] was applied to assess the symptoms of mental health, including somatization disorder, obsessive compulsive symptom, sensitivity to interpersonal relationships, depression, anxiety, hostility, fear, stubbornness, and psychological symptoms. The scale contained 90 items based on a 5-point scale (1, Never; 2, mild; 3, moderate; 4, serious; 5, excessive serious.). A higher score indicated worse mental health status. Respondents with a total score of 160 points, or had more than 43 positive items, or a score of ≥ 2 points for any symptom was considered as having mental problem. The Cronbach’s alpha was 0.954 for this rating scale, and the subscales’ alpha were in order as follows: 0.92, 0.88, 0.89, 0.93, 0.90, 0.85, 0.87, 0.83 and 0.89, respectively.

### Data analysis

All statistical data analyses were performed using Statistical Package for Social Sciences(SPSS) software version 22.0. Descriptive analyses, Chi-square and Independent t-tests were used to compare the socio-demographics and clinical variables between the pediatric nurses with and without HRS. And the Spearman correlation analysis was performed to examine the linear relationships between each independent variable before they entered the model. AMOS 5.0 was used for SEM analysis, and this SEM model estimation was conducted using maximum-likelihood estimation. Model fit indices were examined to test theoverall fit of the model to the collected data including the chi-square test, the goodness-of-fit index(GFI; values approximating 0.90), the comparative fit index (CFI; values approximating 0.90), and the root mean square error of approximation (RMSEA; values approximating 0.08). In all analyses, statistical significant was set as a two-tailed p-value of < 0.05.

## Results

### Participant characteristics

Among the 399 questionnaires delivered, 381 valid questionnaires were returned (response rate 95.5%). There were 25(6.6%) male nurses and 356(93.4%) female nurses. The age distributions were: < 30 years (*n* = 231, 60.6%); 31–40 years (*n* = 129, 33.9%); and ≥ 41 years (*n* = 21, 5.5%). There were 98(25.7%) nurses from Department of Internal Medicine, 107 (28.1%) nurses from Department of Surgery, 78(20.5%) nurses from Department of Outpatient and Emergency, and 98(25.7%) nurses from the ICU. 44(11.6%) nurses had a college degree, 331(86.9%) had a bachelor’s degree, and 6 (1.6%) had a master’s degree.

### Study factors associated with Occupational Stress among pediatric nurses

For whole sample, the mean score of CPSS was 25.80(SD = 6.85), and approximately 54.3% were reported as having HRS. The comparison of socio-demographic factors and the related clinical characteristics between the enrolled patients with and without HRS was displayed in Table 1. We found no significant difference in the following demographic variables: sex, age, education attainment, work department, or work experience between the two groups (all p > 0.05), but the comparison of medical professional title showed a significant p value (*p* = 0.041). In addition, the two groups of pediatric nurses presented significantly different style of coping and profiles of mental health symptoms, such as obsessive–compulsive symptoms, depression, hostility, sensitivity to interpersonal relationships.

**Table 1:** Characteristics of pediatric nurses with and without HRS (*N* = 381)

Variables	Without HRS N(%)/Mean(SD)	With HRS N(%)/Mean(SD)	*X*^*2*^*/t*	*P*
Department			4.981	0.173
Internal Medicine	41(41.84)	57(58.16)		
Surgery	42(39.25)	65(60.75)		
Outpatient and Emergency	41(52.56)	37(47.44)		
ICU	50(51.02)	48(48.98)		
Sex			2.215	0.137
Male	15(60.00)	10(40.00)		
Female	159(44.67)	197(55.34)		
Age(years)			2.894	0.235
≤ 30	100(43.29)	131(56.71)		
31–40	61(47.29)	68(52.71)		
≥ 41	13(61.90)	8(38.10)		
Education attainment			0.137	0.934
College degree	21(47.72)	23(52.27)		
Bachelor’s degree	150(45.31)	181(54.68)		
Master’s degree	3(50.0)	3(50.0)		
Medical professional title			**6.402**	**0.041**
junior	26(46.43)	30(53.57)		
intermediate	116(42.49)	157(57.51)		
senior	32(61.54)	20(38.46)		
Mental Health				
somatization disorder (≥ 2 scores)	29(26.61)	80(73.39)	**22.363**	**0.000**
obsessive compulsive symptom (≥ 2 scores)	64(32.32)	134(67.68)	**29.592**	**0.000**
sensitivity to interpersonal relationships(≥ 2 scores)	28(23.93)	89(76.07)	**32.156**	**0.000**
depression (≥ 2 scores)	29(23.02)	97(76.98)	**38.936**	**0.000**
anxiety (≥ 2 scores)	19(21.59)	69(78.41)	**26.738**	**0.000**
hostility (≥ 2 scores)	28(22.40)	97(77.60)	**40.597**	**0.000**
fear(≥ 2 scores)	19(29.69)	45(70.31)	**7.918**	**0.005**
stubbornness(≥ 2 scores)	20(25.32)	59(74.68)	**16.639**	**0.000**
psychological symptoms(≥ 2 scores)	19(24.05)	60(75.95)	**18.773**	**0.000**
Coping style(scores)				
positive coping style	2.08(0.45)	1.69(0.48)	**-8.334**	**0.000**
negative coping style	1.13(0.48)	1.22(0.50)	1.884	0.600

*HRS* Health related stress

### Correlation matrix for Mental Health, Occupational Stress and Coping Style among pediatric nurses

Table 2 displayed the results of the Spearman correlation. A significantly positive correlation existed between occupational stress and the status of mental health *(all P* < *0.01)*. As for coping style, positive coping style had a significantly negative correlation with the presence of mental health symptoms *(all P* < *0.01)*, whereas negative coping style had a significantly positive correlation *(all P* < *0.01)*. More specifically, the severity of mental health symptoms increased (e.g. obsessive–compulsive symptoms, depression, hostility, sensitivity to interpersonal relationships) as the more negative coping style taken and decreased as the taking positive coping style.

**Table 2:** Correlation matrix for main study variables (*N* = 381)

Variables	Somatization disorder	Obsessive–compulsive symptom	Sensitive to interpersonal relationship	Depression	Anxiety	Hostility	Fear	Stubbornness	Psychological symptoms	Total scores
CPSS	0.416^**^	0.467^**^	0.440^**^	0.527^**^	0.476^**^	0.476^**^	0.323^**^	0.388^**^	0.385^**^	0.492^**^
Coping style										
Positive coping style	-0.262^**^	-0.191^**^	-0.257^**^	-0.305^**^	-0.272^**^	-0.291^**^	-0.216^**^	-0.254^**^	-0.250^**^	-0.285^**^
Negative coping style	0.189^**^	0.223^**^	0.211^**^	0.212^**^	0.212^**^	0.229^**^	0.266^**^	0.251^**^	0.194^**^	0.237^**^

^*^P < 0.05, **P < 0.01

### The Structural Equation Model of occupational stress, coping style and mental health

Our model showed that the overall fit information was as follows: X^2^ = 3.299; GFI = 0.925, CFI = 0.974, and RMSEA = 0.078. These indicators proved to fit the data adequately based on the standards of model testing. Moreover, we found that positive coping style had a negative direct predictive effect on occupational stress (*β* = *-0.499, P* < *0.01*) and mental health symptoms (*β* = *-0.115, P* < *0.01)*, negative coping styles had positive predictive effect on occupational stress (*β* = *0.185, P* < *0.01*) and mental health symptoms (*β* = *0.205, P* < *0.01*), and occupational stress had significant impact on mental health symptoms (*β* = *0.416, P* < *0.01*). We also found that occupational stress was played a part of mediating effect between coping style and mental health symptoms, with a mediating effect of 64.33%, 27.24% for positive coping style path and negative coping style path, respectively.

## Discussion

Overall, our study revealed that 54.3% of the pediatric nurses reported having health risk stress (HRS), and the mean score of CPSS was 25.80(SD=6.85), which was much higher than that among the radiological medical personnel in Zhengzhou, China (22.11(SD=4.69)) [33] and among the medical personnel in Sichuan, China (25.31 (SD =6.44)) [34]. The results of the present study regarding the CPSS test revealed that pediatric nurses working in locked units experienced relatively high levels of stress. Such reports were similar to previous studies conducted in Korea [35] and the Iran [36] though the evaluation tools were using differently. It is may related to the working conditions of pediatric nurses have become exhausting due to service particularity, heavy workload, occupational environment and complexly interpersonal relationships, work-family conflicts, notable is the high prevalence of aggressive behavior and workplace violence towards nurses, as stated in the quotes.

With respect to the degree of occupational stress of nurses in accordance with their general characteristics, nurses with intermediate-professional title had a higher degree of stress than those with entry-level or senior-level groups. This result was similar to the findings of a study conducted by Han and Yu [37], the highest number of intermediate nurses, the greater the work demands. Specifically, the intermediate group with three to five years of experience typically has high work demands because of administrative work along with basic and more advanced tasks, such as being a preceptor or researcher [38]. For example, little or rarely experience in scientific research and paper writing means rarely chances to promote further development, which becomes a main work- stressor and a big barrier for most of nurses in China [28, 39, 40]. But previous studies [12, 28, 38] have carried out the socio-demographic characteristics factors related to stress were following as gender, age, average daily working hours, position, period of shift working experience et al. The discrepancy between the reported studies and ours may result from the differences in measures and sample composition.

Pediatric nurses are the major force in hospitals and are in the front line in contact with ill children and their parents. Suspicion of child abuse and critically ill children are two topics that distinguish pediatric from other specialties and why this specific specialty can have high stress and emotional burden [9]. In our study, we found that a significantly positive correlation existed between the presence of mental health symptoms (e.g., obsessive-compulsive symptoms, depression, hostility, or sensitivity to interpersonal relationships) and occupational stress in pediatric nurses. The degree of occupational stress was higher among participants that responded with “symptoms” as their subjective mental health status. This result was most likely affected by the lack of workplace support or low job control because of the repetitive tasks, low rewards or poor relationships with colleagues, illness children and their families. With the damages to mental health symptoms, it could hinder work performance and the ability to copy with daily work life [41], but more health of nurses’ s mental health or rarely occur psychological symptoms, the better they well deal with job related stressors [42]. In addition, pediatric nurses who perceived stress was more likely to report having mental health symptoms. It was confirmed in some previous study [42, 43] that occupational stress was an important influential factor for nurses’ mental health. Then, it could be concluded that there is an interactive relationship between the two variables. Furthermore, positive coping style had a significantly negative correlation with the presence of mental health symptoms, whereas negative coping style had a significantly positive correlation. Emotional eating, for example, characterized by overeating in response to negative mood states, is a type of altered eating behavior that modifies preferences and consumption patterns and represents a type of dysfunctional coping strategy [44]. Unfortunately, it has been shown that the prevalence of emotional eating was high in nurses, with frequent association with mental health status, such as depression and anxiety [45]. It was worth mentioning that nurses who engaged in emotional eating generally did not have the capacity for adequate coping strategies for negative emotions, a situation that frequently triggered higher food intake.

**Fig. 1:**
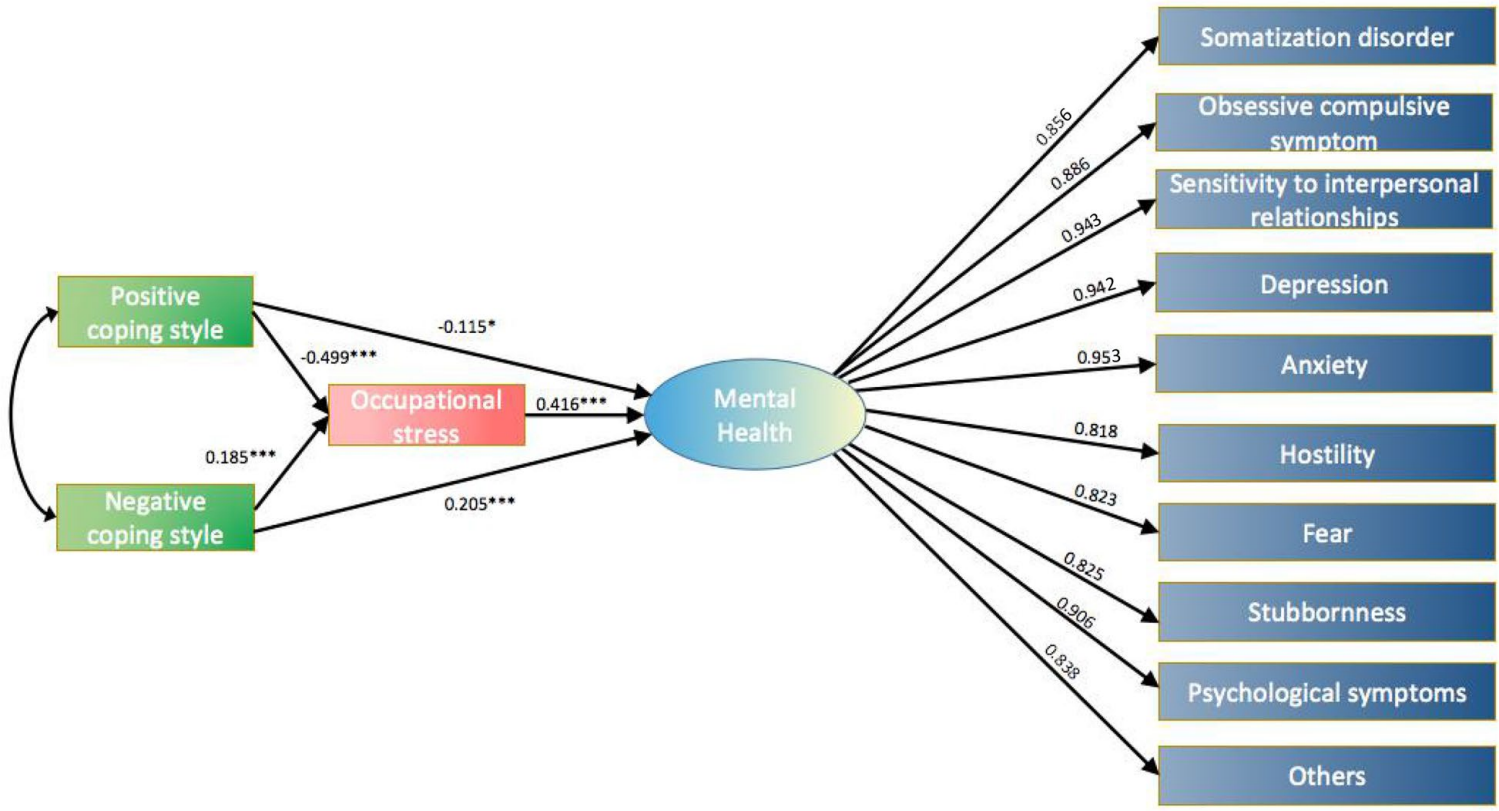
Graphical research model for SEM (Structural Equation Modelling) analysis

Looking at the level of mental health among our participants, the study revealed further significant relationship among these variables. Changes on some variables will predict changes on other variables. From the SEM model in Fig. 1, it could be seen that coping styles was an important independent variable that have several paths of influence with other variables in the model. For instance, positive coping style had a negative direct predictive effect on occupational stress (-*0.499*) and mental health symptoms (*-0.115*), negative coping style had positive predictive effect on occupational stress (*0.185*) and mental health symptoms (*0.205*). Consistently, Hasan et al. [46] illustrated that strategies for coping were inversely correlated with work stress and depression. Alhadi HA [47] found that effective utilizing of coping mechanism interferes with the experienced level of stress and mental health symptoms. This means that a stress level and mental health symptoms could be reduced considerably if an individual knows how to cope with stressors. A nurse who used ineffective coping techniques such as secrecy was at higher risk of psychological distress than that who used more effective coping strategies [48]. It could be explained by different stressful situations and mood experiences might required different, multiple, and complexly coping styles. Empowering nurses may have increased self-efficacy and reduced the occupational stress experienced by improving coping strategies, the positive coping style beneficial for nurses to improve the psychological adaptability, while the negative style would aggravate physical and mental exhaustion [48, 49]. There can, however, be variability with an individual’s ability to cope over time with mental demands and thus propagate higher stress levels [12]. Moreover, occupational stress had significant impact on mental health symptoms (*0.416*), this relationship reflected that the level of work-related stress increased, the presence of obsessive–compulsive symptoms, depression, hostility, anxiety, and so on also increased, consistent with previous research [5, 46]. It seems that, increased levels of occupational stress would result in changes in body systems, including the endocrine system, which may consequently reduce the individual’s capacity of social adaptation and mental health [50]. Furthermore, we found that occupational stress played a part of mediating effect between coping style and mental health. It means that coping styles could indirectly affect the mental health of pediatric nurses through occupational stress. Doerhoff R et al. [12] have also reported that occupational stress could lead to emotional exhaustion, poor-quality sleep due to maladaptive coping strategies (such as high fat diet, alcohol, and smoking). Thus, future intervention programs should target pediatric nurses (e.g., establish an accessible employee assistance program for then) as well as their mental health, especially to decrease the work-related stress problems and enhance positive coping style. Doing this can strengthen work satisfaction in clinical setting and reinforce better coping strategies. This study also has some limitations. Firstly, the study data was obtained from a single city in southwestern China, the sample may not have been representative of Chinese pediatric nurses. Future research could increase the sample size such as involving the nurses from different grades or regions of hospital. Second, the study is mainly used structure questionnaires. So that, qualitative study approach is recommended to explore this issue further. Thirdly, we gave little consideration on the relations between occupational stress, coping styles and mental health, which need to be further researched in the future.

## Conclusion

Despite these limitations, this study revealed significantly associations between the profiles of mental health symptoms and increased occupational stress, and differently coping style had significant differently impact on mental health symptoms (e.g., obsessive-compulsive symptoms, depression, anxiety) in pediatric nurses. Additionally, we identified that occupational stress and coping styles were important predictors for mental health symptoms, and occupational stress played a part of mediating role between coping style and mental health symptoms. The present study provided valuable insights into the correlation among occupational stress, coping styles and mental health, and may offer guidance for develop a better support system or implement ways to cope with this issue for pediatric nurses.

## Data Availability

The datasets used and analysed during the current study are available from the corresponding author(Huaying Yin) on reasonable request.

## Abbreviations

CPSS: A Chinese version of Perceived Stress Scale
HRS: Healthy risk stress
